# Healthcare worker access to molnupiravir: A case series

**DOI:** 10.1371/journal.pone.0282695

**Published:** 2023-03-14

**Authors:** Jessica C. O’Keeffe, Meg Constable, Janice Chiang, Margaret Somerville, Arvind Yerramilli, Ross Nolan, Greg Weeks, Daniel P. O’Brien

**Affiliations:** 1 Department of Infectious Diseases, Barwon Health, Geelong, Victoria, Australia; 2 Barwon South West Public Health Unit, Barwon Health, Geelong, Victoria, Australia; 3 StaffCare, Barwon Health, Geelong, Victoria, Australia; 4 Pharmacy Department, Barwon Health, Geelong, Victoria, Australia; Carol Davila University of Medicine and Pharmacy, ROMANIA

## Abstract

Molnupiravir, an oral antiviral shown to reduce COVID-19 severity, is available in Australia via the Pharmaceutical Benefits Scheme (PBS) for treatment of mild-moderate COVID-19. For people less than 70 years of age it is only available with risk factors for severe disease, hence the majority of healthcare workers do not qualify. Currently, Australian health services are under considerable strain due to COVID-related staff shortages. Thirty staff members of a tertiary hospital, not eligible under the PBS, were offered molnupiravir within the first five days of COVID-19 illness. Their median age was 43 years, and 73% were female. All completed treatment with rates of adverse events that were low and comparable with clinical trial data. The reported duration of illness ranged from 1–16 days with a median of four days. A negative rapid antigen test on the final day of treatment was reported in 81% of people, and 73% reported being well enough to return to work at the completion of mandatory isolation. Only 22% of people reported transmission in their household after they commenced treatment. The implementation of a policy allowing access to molnupiravir outside of PBS recommendations for healthcare workers with mild-moderate COVID-19 may have important individual benefits to workers health and wellbeing and help alleviate the acute staff shortages experienced currently by the Australian healthcare workforce.

## Introduction

Molnupiravir (Lagevrio®) is an oral antiviral currently available in Australia via the Pharmaceutical Benefits Scheme (PBS) for the treatment of mild-moderate Coronavirus 19 Disease (COVID-19) in people with risk factors for severe disease [1]. When commenced within five days of symptom onset, it has been shown to reduce the risk of hospitalisation and death as well as symptom duration and the need for respiratory interventions or acute healthcare visits in those with risk factors for more severe disease [2–5]. Additionally, it may reduce illness severity and duration and visits to primary care in those without identified risk factors for severe disease [6–8]. There is also emerging evidence that oral antiviral treatment may reduce the risk of long-COVID [9]. It may also reduce infectiousness and transmission by rapidly reducing viral load [6–8, 10]. In published studies it has been very well tolerated with adverse effect profiles equivalent to placebo [2, 6–8]. It is orally administered, has no known drug interactions and does not require dose adjustments in those with renal or hepatic dysfunction. Due to concerns about teratogenicity, it is not recommended for use during pregnancy or lactation, and effective contraception is recommended for women of childbearing age during and for four days after treatment and men with a partner of childbearing potential during and for three months after treatment [11].

The Australian healthcare system is under immense pressure from COVID-19, including ongoing staff absences as a result of sick leave, further complicated by burnout from heavy workloads throughout the pandemic. Thus it is important to support the health and wellbeing of healthcare workers and their households. This could be achieved by efforts to reduce the duration and severity of their illness, the burden of illness in their households, as well as make them feel valued, safe and motivated at work. This will in turn help protect the healthcare system. As the majority of healthcare workers (HCWs) do not qualify for antiviral therapy via the PBS, in order to support its workforce for the above reasons, molnupiravir was made available outside of the PBS recommendations to HCWs at a tertiary healthcare facility in Victoria, Australia for a limited period during a predominantly Omicron BA.2 wave of COVID-19 with approval from the Victorian Department of Health.

This case series reports the impact of policy implementation by describing the uptake, tolerability, duration and severity of illness, further infections in the household, and time off work among staff who were prescribed molnupiravir after becoming mildly unwell with COVID-19 and who did not qualify for PBS listed treatment. Molnupiravir was taken voluntarily outside of PBS recommendations with informed consent through the internal staff medical service.

## Methods

The availability of molnupiravir was communicated to staff by their managers and through online staff communications. Those who were within five days of symptom onset, not eligible for PBS funded antiviral therapy, and interested in taking molnupiravir were directed to the internal staff medical service (StaffCare) for assessment. From June 20^th^ to 28^th^ 2022, 30 staff members voluntarily received molnupiravir at a dose of 800mg twice daily for five days. Staff were required to not be pregnant or breastfeeding based on history, and if relevant, be able to comply with recommended contraception for the specified time [11]. Staff COVID-19 vaccination status was not collected, but it was a legislative requirement at the time for all staff to have received three doses of Therapeutic Goods Administration approved COVID-19 vaccine.

When receiving the medication all staff were provided with rapid antigen tests (RATs), and asked to perform a RAT on their final day of treatment, and to test other household members at the time treatment commenced. A follow-up survey was emailed to participants two weeks after treatment commenced and information gathered was stored in a secure RedCap database. An individualised survey link was used, allowing the investigator to monitor who had completed the survey, though the survey was completed in a de-identified manner, such that results could not be traced back to an individual. Data collected included basic demographics, co-morbidities, symptom severity and duration, medication related adverse effects, and time off work. Staff were asked to rate their symptom severity on a scale of 1–10 (1 = mild, 10 = severe). They were asked to report the RAT result performed on day five of treatment as well as the number of further COVID-19 infections in their household members. They were also asked to rate their satisfaction with the program.

This case series received research and ethics approval from the Barwon Health Ethics Committee, approval number HREC/88780/VICBH-2022327587. All participants provided written informed consent for their information to be presented for publication.

## Results

Twenty-two (73%) participants were female, with an age range of 21–69 years (median 43 years). Most (80%) reported no co-morbidities. The majority were nurses (50%) or doctors (24%) (Table 1). All participants completed the five-day course of molnupiravir, and none reported any difficulties accessing the medication. There were eight (27%) reports of mild adverse events including diarrhoea (13%), dizziness (10%) and nausea (7%) (Table 2). No severe adverse events or hospitalisations were reported.

**Table 1:** Participant details.

		Number	Percentage (Total = 30)
**Age (years)**		21–69	Median: 43
**Female**		22	73%
**≥1 Co-morbidity**	None	24	80%
	Hypertension	3	10%
	Ischaemic Heart disease	1	3%
	Asthma	2	6%
	Diabetes Mellitus	1	3%
**Day of Illness**		0–4	Median: 1
**Occupation**			
	Doctor	7	24%
	Nurse	15	50%
	Patient Service Assistant	1	3%
	Allied Health	3	10%
	Administration	3	10%
	Other	1	3%

**Table 2:** Outcomes.

		Number	Percentage (Total = 30)
Adverse Events			
	None	22	73%
	Diarrhoea	4	13%
	Dizziness	3	10%
	Nausea	2	7%
No. of people with issues accessing the drug		0	0%
No. of people self-reporting completing treatment course		30	100%
No. of people with further COVID-19 cases in the household after treatment commenced (Total = 27 participants with other people in the house)		6	22%
	<48hrs after commencing treatment	3*	10%
	>48hrs after commencing treatment	2*	7%
No. of people who performed a RAT on day 5 of treatment		27	90%
No. of people who had a NEGATIVE RAT on day 5 of treatment		22	73%
No. of people who reported being fit to return to work by day 7		22	73%

*One person did not report the time frame in which household members became positive.

**Fig 1:**
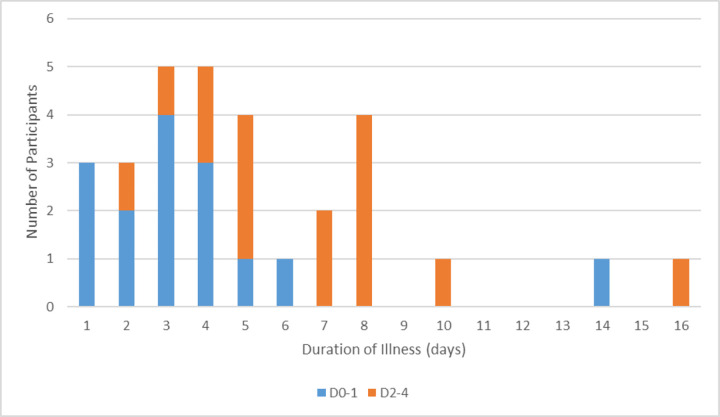
Duration of illness by day of molnupiravir prescription. Breakdown of duration of COVID-19 illness by day in which antiviral therapy was commenced. Cases divided into either D0-1 (received antiviral therapy on Day 0 or Day 1 of symptoms) or D2-4 (received antiviral therapy on Day 2, Day 3 or Day 4 of symptoms). NB: Day 0 is the first day of symptom onset.

The reported duration of illness ranged from 1–16 days with a median of four days (Fig 1). Overall, reported illness severity ranged from 1–9, with a median of five, with all participants reporting an improvement in symptoms within 48-hours of treatment commencing. A RAT was performed by 27 people on the final day of treatment, and was reported as negative in 81% of cases (22/27). Of the 27 people who resided with others, only six (22%) reported additional household members testing positive after treatment commenced, with most testing positive within 48-hours of treatment commencing (Table 2). Twenty-two (73%) reported feeling well enough to return to work at the end of their mandatory isolation period of seven days from their initial positive COVID-19 test (RAT or PCR). No participants reported a recurrence of symptoms in the 14-days post start of treatment.

Half the participants received their antiviral therapy within 48-hours of symptom onset (D0-D1). For these the median duration of illness was reported to be three days, and of those who tested, the rate of a negative RAT on day five was 93% (13/14). Furthermore, 79% (11/14) of these were asymptomatic with a negative RAT by day five of treatment (Table 3).

**Table 3:** Outcomes based on time to molnupiravir commencement from symptom onset.

Day of antiviral treatment from symptom onset	No. RAT Negative at day 5 of treatment*	%	No. Asymptomatic at Day-5 from symptom onset^#^	%	No. of RAT Negative AND Asymptomatic at Day-5	% (Total = 27, D0-1 = 14, D2-4 = 13)	% (Total = 30, D0-1 = 15, D2-4 = 15)
**D0-1**	13	93%	13	87%	11	79%	73%
**D2-4**	9	69%	7	47%	5	38%	33%
**Total**	22	81%	20	67%	16	59%	53%

*No. of people who performed a RAT on Day-5 total = 27, D0-1 = 14, D2-4 = 13.

^#^No. of people who were asymptomatic: Total population = 30, D0-1 = 15, D2-4 = 15.

One hundred percent of participants reported being satisfied (6/30) or extremely satisfied (24/30) with the program, and many independently sent messages to those implementing the program, expressing appreciation at being given access to the medication.

## Discussion

The healthcare system in Australia continues to be under immense strain, substantially contributed to by high levels of COVID-19 related sick leave and unfilled vacancies due to staff burnout. Additionally, HCWs from all disciplines have worked tirelessly over the COVID-19 pandemic, with risks to their own safety and that of their family members, resulting in great benefit to the community. Supporting HCWs health and wellbeing, and helping to ensure that they feel safe, valued, supported and motivated is crucial to retaining the current workforce and healthcare capacity.

We feel the experience of offering access to the antiviral molnupiravir at a large tertiary centre has made a significant impact in supporting its healthcare workforce. Uptake and acceptance of the treatment was high (30 participants in nine days), participants reported high levels of satisfaction with the program and were very appreciative of its availability, and all participants completed the treatment without any severe adverse effects reported.

As this was not a placebo-controlled trial, we cannot definitively determine the effectiveness of molnupiravir treatment on symptom severity and duration, however it is promising to note all participants reported an improvement in symptoms within 48-hours of commencing treatment, and the median duration of symptoms was reported as four days, and three days for those commencing treatment on D0-1 of symptoms, as compared to a published average duration of illness with the Omicron strain of 6.87 days [12].

Rapid antigen test results have been correlated with detection of viable virus, and have been proposed as a tool to guide isolation duration policy [13]. Lefferts et al. (2022) reported the rate of negative RATs at day 5–9 of illness in 729 people during the Omicron wave in Alaska. The population was similar to our case series in that it was a highly vaccinated cohort (primary course completion rate 74.2%) with a median age of 30 years (IQR = 17–45). Of the 179 people who were symptomatic and performed a RAT on day five of illness, only 20.7% had a negative result; increasing to 33.3% (74/111) on day seven. This is in contrast to our case series where 93% of those who commenced treatment on D0-1 of symptoms and tested had a negative RAT on day five of treatment.

Many HCWs are concerned about bringing COVID-19 into their households and families, especially if they live with those at higher risk of severe disease. By reducing viral replication it is probable that molnupiravir can also reduce infectiousness and secondary transmission to household members, although this has not yet been shown [8]. The experience in our case series provides some support to this hypothesis, in that 81% of participants had a negative RAT at day five of treatment, but also that there were only two reported cases of further infection in households detected more than 48-hours after treatment commenced with those reported prior to 48-hours likely already infected prior to treatment commencing [14]. This further case rate of 22% is compared with an overall reported household transmission rate of 39% with Omicron BA.2 variants [15]. Furthermore, one of the two reported additional household cases occurred where there was another case positive in the household at diagnosis and thus transmission may have occurred from someone other than the recipient of antiviral therapy.

Maximising the likelihood that people are able to return to work once isolation is complete is vital to maintaining an effective healthcare workforce. From a system perspective, this involves supporting the individual in being well enough to return, but also for those HCWs who are caregivers to others in their household, improving the likelihood that others at home remain disease-free. In a review of HCW sick leave duration by Strum et al. (2022), the mean return-to-work time for unvaccinated and fully vaccinated people was 18.49 and 10.9 days respectively. In our case series, nearly three in four HCWs reported feeling well enough to return to work at the end of their mandatory isolation period (seven days), and as discussed, transmission in the household post treatment commencement appeared low. Furthermore, in the Panoramic trial the median duration of time until first reported recovery was nine days in the group receiving molnupiravir compared to 15 days in those receiving usual care [8]. Therefore access to molnupiravir may help preserve the HCW workforce.

Furthermore, HCW workforce could be even further preserved if release from isolation criteria was reduced. Considering the study by Fischer et al. (2021) that showed virus was not cultured in any participants after five days of molnupiravir [10], if Public Health Authorities agreed to allow return to work at day five of treatment in those who were asymptomatic, received five days of molnupiravir and had a negative RAT on day five of treatment; up to 79% of participants who received treatment by D1 of symptoms in our case series could have returned to work earlier than the currently mandated isolation period.

When reviewing the feasibility of implementing widespread use of molnupiravir in this manner, broader public health considerations must also be addressed. Molnupiravir was provided free of charge to participants. Given the cost of treatment (approximately $A1000 per course) unless subsidised stock is made more widely available through the PBS scheme or the National Medicines Stockpile, access will remain problematical for the majority of health care workers. Sanderson et al. (2023) raises a potential concern regarding its widespread use, and their work remains an ongoing area of research. The mechanism of action of molnupiravir is to induce mutations that have a deleterious effect on the virus, though this raises the possibility that molnupiravir could contribute to the development of further mutations and potentiate variants of concern [16].

There are limitations to our case series findings including the small numbers included and the lack of a comparator group. Data on household transmission including identification of the index case, and the exact number of subsequent infections was not collected. However, given the overall positive findings, we would advocate for further evaluation of this intervention in a larger population of HCWs.

In conclusion, the implementation of a policy that allows access to molnupiravir outside of PBS recommendations for HCWs with mild-moderate COVID-19 appeared well accepted and tolerated. It may also have important individual benefits to HCW’s health and well-being and help alleviate the acute staff shortages experienced currently by the Australian health care workforce.

## Supporting information

S1 Table Anonymised raw data set. (XLSX)
